# A novel CapsNet neural network based on MobileNetV2 structure for robot image classification

**DOI:** 10.3389/fnbot.2022.1007939

**Published:** 2022-09-30

**Authors:** Jingsi Zhang, Xiaosheng Yu, Xiaoliang Lei, Chengdong Wu

**Affiliations:** Faculty of Robot Science and Engineering, Northeastern University, Shenyang, China

**Keywords:** robot image classification, CapsNet neural network, MobileNetV2, attention module, spatial and channel information

## Abstract

Image classification indicates that it classifies the images into a certain category according to the information in the image. Therefore, extracting image feature information is an important research content in image classification. Traditional image classification mainly uses machine learning methods to extract features. With the continuous development of deep learning, various deep learning algorithms are gradually applied to image classification. However, traditional deep learning-based image classification methods have low classification efficiency and long convergence time. The training networks are prone to over-fitting. In this paper, we present a novel CapsNet neural network based on the MobileNetV2 structure for robot image classification. Aiming at the problem that the lightweight network will sacrifice classification accuracy, the MobileNetV2 is taken as the base network architecture. CapsNet is improved by optimizing the dynamic routing algorithm to generate the feature graph. The attention module is introduced to increase the weight of the saliency feature graph learned by the convolutional layer to improve its classification accuracy. The parallel input of spatial information and channel information reduces the computation and complexity of network. Finally, the experiments are carried out in CIFAR-100 dataset. The results show that the proposed model is superior to other robot image classification models in terms of classification accuracy and robustness.

## Introduction

In recent years, convolutional neural networks (CNN) have developed rapidly and achieved fruitful results. In terms of military application, convolutional neural network is used to identify and detect military targets (Choi et al., 2020; Yin and Li, 2020; Zeng et al., 2021). However, in the environment of missile-borne terminal, there are high requirements on network and hardware, which requires the network to maintain lightweight and good embedding performance on the basis of ensuring recognition accuracy. After AlexNet achieves a qualitative leap in the classification accuracy of ImageNet large data set (Prabhu et al., 2020), the development trend of convolutional neural network is complicated and the number of convolutional layers is increasing. Representative networks include VGG (Simonyan and Zisserman, 2014) and GoogLeNet (Szegedy et al., 2015), which respectively, study the depth and width of convolutional neural networks and increase the network depth and width to improve the network performance. In 2015, He et al. (2016) innovatively proposed the ResNet network, which introduced the concept of residual for the first time and used residual to transmit information, effectively alleviating the problems of over-fitting and gradient disappearance caused by the increase of network depth. This network also provides ideas for the subsequent development of lightweight. Then, the researchers introduced the attention mechanism into the convolutional neural network. SENet (Hu et al., 2020) in 2017 used the attention mechanism and gave different weights to different feature graphs to improve the learning ability of the network. The later proposed attention modules draw on this idea and improve the SENet.

For example, CBAM module studies spatial attention on the basis of channel attention (Woo et al., 2018). The introduction of this idea makes network design tend to be simple, fast and portable. On the basis of point-by-point convolution and deep convolution, the typical lightweight network MobileNetV2 introduces the backward residual structure and linear bottleneck structure (Sandler et al., 2018), which makes the network structure smaller and the speed further be improved. Branch and Carvalho (2021) combined the MobileNetV2 network model with the traditional Hough transform. It performed better for the identification task, which could improve the accuracy rate with a lower number of parameters and calculation (Shafiq et al., 2021). On the basis of MobileNetV2 network, Yang et al. (2011) drew on the idea of DenseNet dense connection and utilized the benefits of feature reuse to improve network performance and reduce network scale (Akay et al., 2021). Hui et al. (2018) modified the long module on the basis of the bottleneck layer of MobileNetV2, which further reduced the complexity and computation amount when the network accuracy was improved. Cao et al. (2021) chose MobileNetV2 network as the backbone network of target detection and introduced channel attention mechanism to realize feature enhancement and effectively improve the detection performance of the network while ensuring the lightness of the algorithm (Jisi and Yin, 2021). The above methods have the following problems: a lot of network parameters, slow convergence time, easily falling into over-fitting. The CapsNet neural network can save computation time and improve classification accuracy.

Based on MobileNetV2 lightweight network and combined with CapsNet neural network, a channel space dual collaborative attention module is designed in this paper. By giving more weights to the extracted features, the recognition accuracy is increased while ensuring the network lightweight. On the CIFAR-100 standard data set, the effectiveness of the network is verified by comparing different typical convolutional neural networks and different attention modules.

The main structures of this paper are as follows: section Related works displays two networks including MobileNetV2 network and attention mechanism. Section Proposed robot image classification model shows the proposed image classification network. The experiments are conducted in section Experiments and analysis. A conclusion is concluded in section Conclusion.

## Related works

Image classification is widely used in video surveillance analysis, medical image recognition, face image recognition and other fields. Traditional image classification uses artificial design to extract features, but its generalization ability is poor. Deep learning has been successfully used in speech recognition, natural language processing, and especially computer vision. Deep convolutional neural Networks (DCNN) has become a major research method in the field of computer vision. The following introduces two models including MobileNetV2 network and attention mechanism for this paper.

### MobileNetV2 network

With the rapid development of convolutional neural network, its structure is complex, its volume is large, and the calculation amount is large. The demand for hardware resources is bigger. Training cannot be deployed on resource-constrained platforms, so the current research direction is lightweight and high-speed (Zhu et al., 2019). One method is to compress the trained network and get a relatively small model. Another method is to design a small model for training, and MobileNet 2 network is a representative lightweight network.

**Figure 1:**
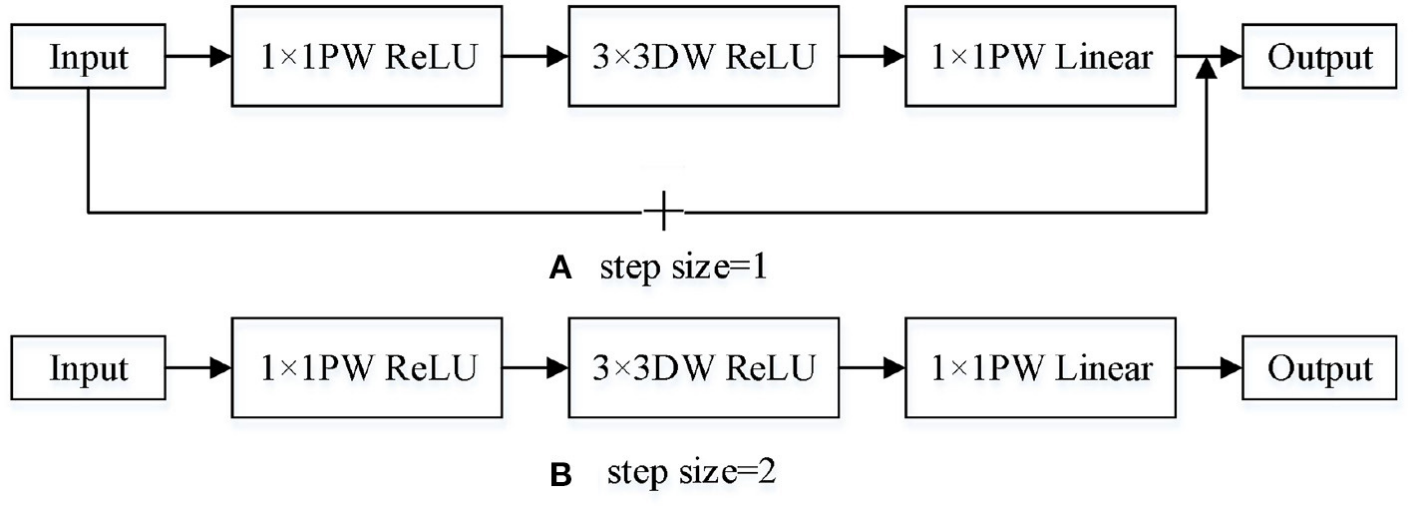
MobileNetV2 structure. **(A)** step size=1. **(B)** step size=2.

MobileNetV2 introduces the reverse residual structure and linear bottleneck structure in the network. The reverse residual structure is different from the traditional residual structure. The traditional residual structure reduces the dimension first and then raises it. The reverse residual structure adopts the opposite order. First, 1 × 1 point-by-point convolution of one layer is used to enhance the dimension, and then 3 × 3 deep convolution is used to replace 3 × 3 standard convolution, which can greatly reduce the amount of computation and improve the effectiveness of the network model. Linear bottleneck structure is to design 1 × 1 point with point convolution to reduce the dimension and add it to the input, remove the activation function ReLU in the last layer and replace it with linear activation function. This method can solve the problem of serious information loss. In addition, expansion coefficients are introduced to control the size of the network. The bottleneck structure is shown in Figure 1.

Table 1 shows the input-output relationship of each layer in the bottleneck diagram in Figure 1. Where k is the number of input channels. h and w are the height and width of the input, respectively. s is the step size. t is expansion coefficient. k' is the number of output channels.

**Table 1:** Relation between input and output.

**Input**	**Operator**	**Output**
*h* × *w* × *k*	1 × 1 PW ReLU	*h* × *w* × (*tk*)
*h* × *w* × (*tk*)	3 × 3 DW ReLU	(*h*/*s*) × (*w*/*s*) × (*tk*)
(*h*/*s*) × (*w*/*s*) × (*tk*)	1 × 1 PW Linear	(*h*/*s*) × (*w*/*s*) × (*k*′)

### Attention mechanism

When the improvement effect of increasing depth and width in convolutional neural network is not obvious, researchers focus on the attention mechanism. The attention mechanism was first applied to natural language processing. In image classification, the attention mechanism makes the convolutional neural network selectively attach importance to some feature images and suppress some feature images by increasing the weight of some feature images. At present, a lot of researches have been carried out in this area. SENet attention designs a channel module, which introduces two processes of compression and excitation, uses global average pooling to compress the space into 1 × 1 × C feature graphs, and then assigns different weights to different feature maps through two fully connected layers (Shafiq et al., 2020; Li et al., 2022). CBAM focuses on spatial attention on the basis of channel attention, and uses global average pooling and global maximum pooling to reset the weight of channel and spatial attention, which proves to be better than single-channel attention mechanism. In addition, CBAM also explores the effect of the order of attention on the classification effect. Recently, the ECANet module was proposed (Wang Q. L. et al., 2020) based on SENet module, it avoided the reduction of channel dimension and achieved the coverage of local cross-channel interaction by increasing the number of one-dimensional convolution, with certain improvement in classification accuracy.

## Proposed robot image classification model

### CapsNet neural network

In deep learning, CNN performs very well in image classification and recognition. However, the internal data of CNN does not consider the important spatial hierarchical relationship among features. Although CNN can reduce the size of data space through the network by maximum pooling or adding subsequent convolutional layers, thus increasing the receptive field of upper neurons, that is, obtaining higher-order features in a larger area of the input image, to a certain extent, the model can improve the ability of image recognition, maximum pooling will lose valuable feature information, and traditional convolutional networks cannot solve the fundamental problem of spatial position relationship between low-level features and high-level features. Sabour et al. (2017) proposed CapsNet neural network based on capsule system and dynamic routing mechanism in 2017, which achieved extremely high classification accuracy in MINST data set. Capsules are used to encapsulate and encode the attributes and spatial relations of features in images, such as position, size, direction, deformation, velocity, reflectivity, color and texture, etc. In the multi-layer capsule system, the lower layer capsule sends it to the higher layer capsule that “agrees” with the output result. This step is realized by the dynamic routing algorithm between capsules (Mobiny and Nguyen, 2018).

### Transmission process between capsules

**Figure 2:**
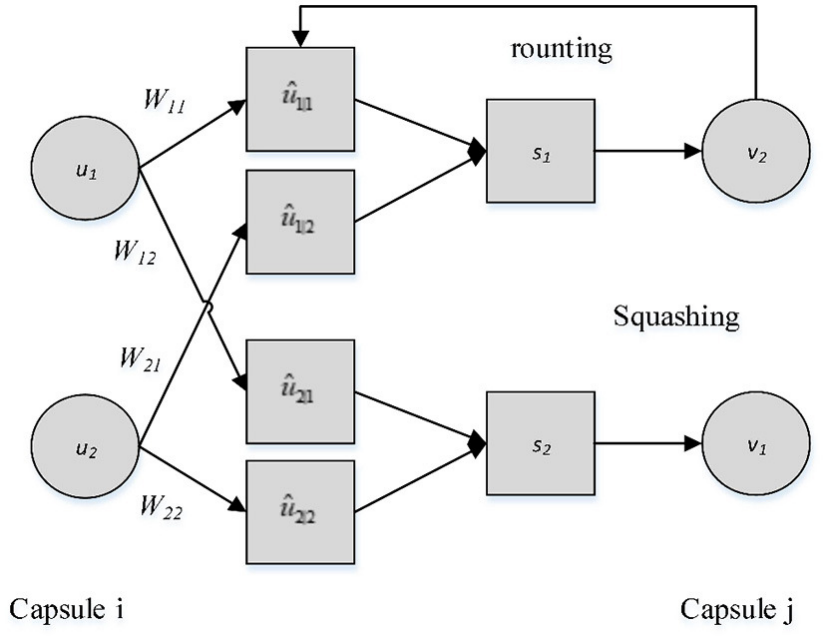
The process of transmission between capsules.

As shown in Figure 2, the input *u*_*i*_ of the CapsNet network model is a vector, which also is a capsule unit. It is similar to the neuron in the convolutional neural network, but the input and output are not scalars but vectors. *u*_*i*_ is multiplied by different weights *W*_*ij*_, respectively, and then it gets the output of the bottom capsule, namely, the prediction vector û_*j*|*i*_. Then û_*j*|*i*_ is multiplied by the corresponding coupling coefficient *c*_*ij*_, namely, it conducts weighted sum to get *s*_*j*_.


(1) $$\begin{array}{l} {û}_{j|i}=W_{ij}u_{i} \end{array}$$

(2) $$\begin{array}{l} s_{j}=\underset{i}{\sum }c_{ij}{û}_{j|i} \end{array}$$

Where, *c*_*ij*_ is the coupling coefficient determined by the iterative dynamic routing process, and there is no bias term in the capsule network model.

The non-linear activation function Squashing is used in CapsNet to scale a vector between 0 and 1. After *s*_*j*_ is obtained, the Squashing function is used to transform vector *s*_*j*_ into vector *v*_*j*_, as shown in Equation (3):


(3) $$\begin{array}{l} v_{j}=\frac{||s_{j}|{|}^{2}}{1+||s_{j}|{|}^{2}}\frac{s_{j}}{\left||s_{j}|\right|} \end{array}$$

Since this function is non-linear, the output *v*_*j*_ preserves the dimension of *s*_*j*_ and does not change the direction of the vector, only the changes the vector size. The length of its output vector represents the probability of a given feature detected by the capsule. Therefore, the probability of each capsule is between 0 and 1.

### Dynamic routing algorithm between capsules

The updating iteration of coupling coefficient *c*_*ij*_ in Capsule network model is realized by dynamic routing algorithm. The input of the dynamic routing algorithm is the prediction vector û_*j*|*i*_ and the number *r* of routing iterations. *b*_*ij*_ is a temporary variable whose value will be updated in the iteration process. When the whole dynamic routing algorithm is finished, its value will be saved to *c*_*ij*_. *b*_*ij*_ is initialized to zero at the beginning of the training (Cao et al., 2020; Madhu et al., 2021; Sepas-Moghaddam et al., 2021).

The following process is iterated *r* times. Firstly, softmax is used to calculate the value of weight *c*_*ij*_, as shown in Equation (4).


(4) $$\begin{array}{l} c_{ij}=\frac{e^{b_{ij}}}{\underset{k}{\sum }e^{b_{ik}}} \end{array}$$

Softmax can ensure that all the weight *c*_*ij*_ is non-negative and the sum is equal to 1.

The weighted sum of û_*j*|*i*_ is then used to obtain *s*_*j*_, and the squashing function is used to obtain *v*_*j*_. The last step of the iteration is to check each high-level capsule input and update the corresponding weight *b*_*ij*_.


(5) $$\begin{array}{l} b_{ij}\leftarrow b_{ij}+{û}_{j|i}\cdot v_{j} \end{array}$$

After updating the weights, the algorithm returns and restarts the calculation of *c*_*ij*_, and repeating *r* times. Dynamic routing algorithm is easy to converge, but it also has the problem of over-fitting. Although increasing the number of iterations can improve the accuracy, it will increase the generalization error, so it is not suitable for too many iterations.

Two methods are used to optimize and improve dynamic routing. One is to introduce Adam function, the other is to improve the compression function. Shafiq et al. (2020) added Adam algorithm in the dynamic routing, which could make the model convergence more stable. The algorithm averages the moving index of the parameter error between capsules, dynamically adjusts the Leaning Rate of the routing algorithm, and adjusts the weight *b*_*ij*_.

In this paper, Adam algorithm is optimized, and dynamic routing is improved. During iteration, Sigmoid after translation is multiplied with *s*_*j*_/||*s*_*j*_|| to replace Squashing function, and Squashing function is used in the final output. The improved algorithm is as follows as shown in Algorithm 1:

**Algorithm 1:** Modified dynamic routing.

Procedure *I* − *ROUTING*(û_*j*|*i*_, *r, l*) for all capsule *i* in layer *l* and *j* layer (*l*+1) for r iterations do (6) $$\begin{array}{l} c_{i}\leftarrow soft\max\left(b_{i}\right) \end{array}$$ (7) $$\begin{array}{l} {\overset{⌢}{m}}_{t}\leftarrow m_{t}/\left(1-\alpha^{t}\right) \end{array}$$ (8) $$\begin{array}{l} {\overset{⌢}{n}}_{t}\leftarrow n_{t}/\left(1-\beta^{t}\right) \end{array}$$ Output: return squash (*v*_*j*_)

For each category k appearing in the image, the capsule uses a separate profit loss *L*_*k*_, as shown in Equation (6).


(9) $$\begin{array}{l} L_{k}=T_{k}\max{\left(0,m^{+}-||v_{k}||\right)}^{2}+\lambda \left(1-T_{k}\right)\max{\left(0,||v_{k}||-m^{-}\right)}^{2} \end{array}$$

If there are k images, it sets *T*_*k*_ = 1, and *m*^+^ = 0.9, *m*^−^ = 0.1. The value of λ is 0.5 to reduce the loss when some categories do not appear, so as to ensure that the vector modulus representing the digital capsules of this category is as large as possible, and the vector modulus of other categories is as small as possible to achieve accurate classification. The total loss is the sum of all the digital capsule losses.

The definition of softmax function is given by formula (4), the definition of Squashing function is given by formula (3), and the Sigmoid function is as follows:


(10) $$\begin{array}{l} sigmoid\left(\left||s_{j}|\right|\right)=1/\left(1+e^{-||s_{j}||}\right) \end{array}$$

Because CapsNet network finally measures the probability of a result by the modulus size of the vector. The magnitude of the modulus is directly related to the probability. The larger modulus denotes the greater corresponding probability. The Sigmoid function converges faster than the Squashing function. Therefore, using the translation Sigmoid function multiplied with *s*_*j*_/||*s*_*j*_|| to replace the Squashing function in the iterative link can play an amplification effect when the result is close to 0 or 1. So it can improve the recognition accuracy of some specific kinds of images, and improve the efficiency of model operation.

### CapsNet based on MobileNetV2 network

In view of the requirements of image classification for the lightweight and accuracy of deep convolutional neural network, this paper takes the lightweight network MobileNetV2 as the benchmark, and then outputs the feature map from CapsNet into MobileNetV2. On the basis of ensuring the lightweight, the self-designed attention mechanism structure is added to improve the classification accuracy of the network. In the process of channel attention and spatial attention, small convolution blocks are used to ensure the computational requirements of the network. Furthermore, convolutional blocks of different sizes are designed to extract multi-scale information of feature graphs, and convolutional blocks with different dilated rates are designed to make the network pay attention to global features. Finally, it adds the designed attention module to the MobileNetV2 network structure.

**Figure 3:**
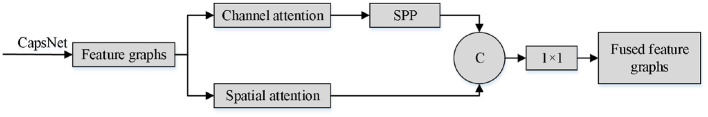
Proposed C-MobileNetV2 structure.

A new image classification structure C-MobileNetV2 is proposed in this paper, and its structure is shown in Figure 3. First, it inputs the feature graphs extracted by CapsNet into MobileNetV2. Feature graphs enter the channel and spacial modules in parallel. Then, the multi-scale semantic information is extracted through the spatial pyramid pooling (SPP) layer (Tai et al., 2020; Wang Q. L. et al., 2020) in the channel attention module and enters into the multi-layer perceptron. Under the action of activation function, the feature graph after redistributing weight is obtained. In the spatial attention module, the convolution of different scales is designed to increase the receptive field, and the spatial feature map with comprehensive semantic and distinct characteristics is obtained. Finally, the feature graphs generated by the two modules are fused to generate the extracted feature graphs.

Because MobileNetV2 introduces the reverse residual structure and linear bottleneck structure, the computational cost is greatly reduced. This paper introduces self-designed attention module to improve its classification accuracy. The improved network structure is shown in Table 2. The designed attention module is integrated into the third, fifth, sixth, and seventh layers of the network structure. The purpose of the design is to increase the recognition accuracy without changing the size of the network and the amount of computation.

**Table 2:** Parameters in C-MobileNetV2 structure.

**Layer**	**Input**	**Operator**	**t**	**c**	**n**	**s**	**C-MobileNetV2**
Input layer	224 × 224 × 3	conv2d	–	64	1	2	×
1	112 × 112 × 34	Bottleneck	1	16	1	1	×
2	112 × 112 × 16	Bottleneck	6	24	2	2	×
3	56 × 56 × 24	Bottleneck	6	32	3	2	√
4	28 × 28 × 32	Bottleneck	6	64	4	2	×
5	14 × 14 × 64	Bottleneck	6	96	3	1	√
6	14 × 14 × 96	Bottleneck	6	160	3	2	√
7	7 × 7 × 160	Bottleneck	6	320	1	1	√
8	7 × 7 × 320	conv2d 1 × 1	–	1,280	1	1	–
9	7 × 7 × 1,280	Avgpool 7 × 7	–	–	1	–	–
Output layer	1 × 1 × 1,280	conv2d 1 × 1	–	100	–	–	–

#### Channel attention module

**Figure 4:**
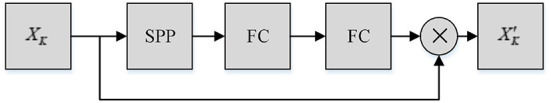
Channel attention.

Channel attention module is shown in Figure 4. When the input feature graph $X_{K}\in R^{H\times W\times C}$ is entered, Through spatial pyramid pooling, global information is compressed by H × W in spatial dimension. The channel feature graph is compressed into 1 × 1 × C, and the corresponding weight information is generated by mapping. The spatial pyramid adaptively and evenly stores the input feature graphs into three scales. The output of the three channels is adjusted to three one-dimensional vectors, and the one-dimensional attention graph is generated after fusion.

In order to assign different weights to different channels, a one-dimensional attention diagram is extracted from the pooling layer of spacial pyramid. The two fully connected layers are followed by the sigmoid function to normalize the output to the range of (0,1). The output after SPP pooling layer and two full connection layers is:


(11) $$\begin{array}{l} \tilde{M}=sig\left(W_{2}\rho \left(W_{1}M\right)\right) \end{array}$$

Where $\tilde{M}$ is the output after SPP pooling layer and two fully connection layers. *W*_1_ and *W*_2_ are the fully connected layer 1 and layer 2, respectively. M represents the one-dimensional feature graph generated after passing through the SPP layer. ρ denotes ReLU.

The output of spatial channel attention mechanism is:


(12) $$\begin{array}{l} X_{K}=X_{K}⊗\tilde{M} \end{array}$$

**Figure 5:**
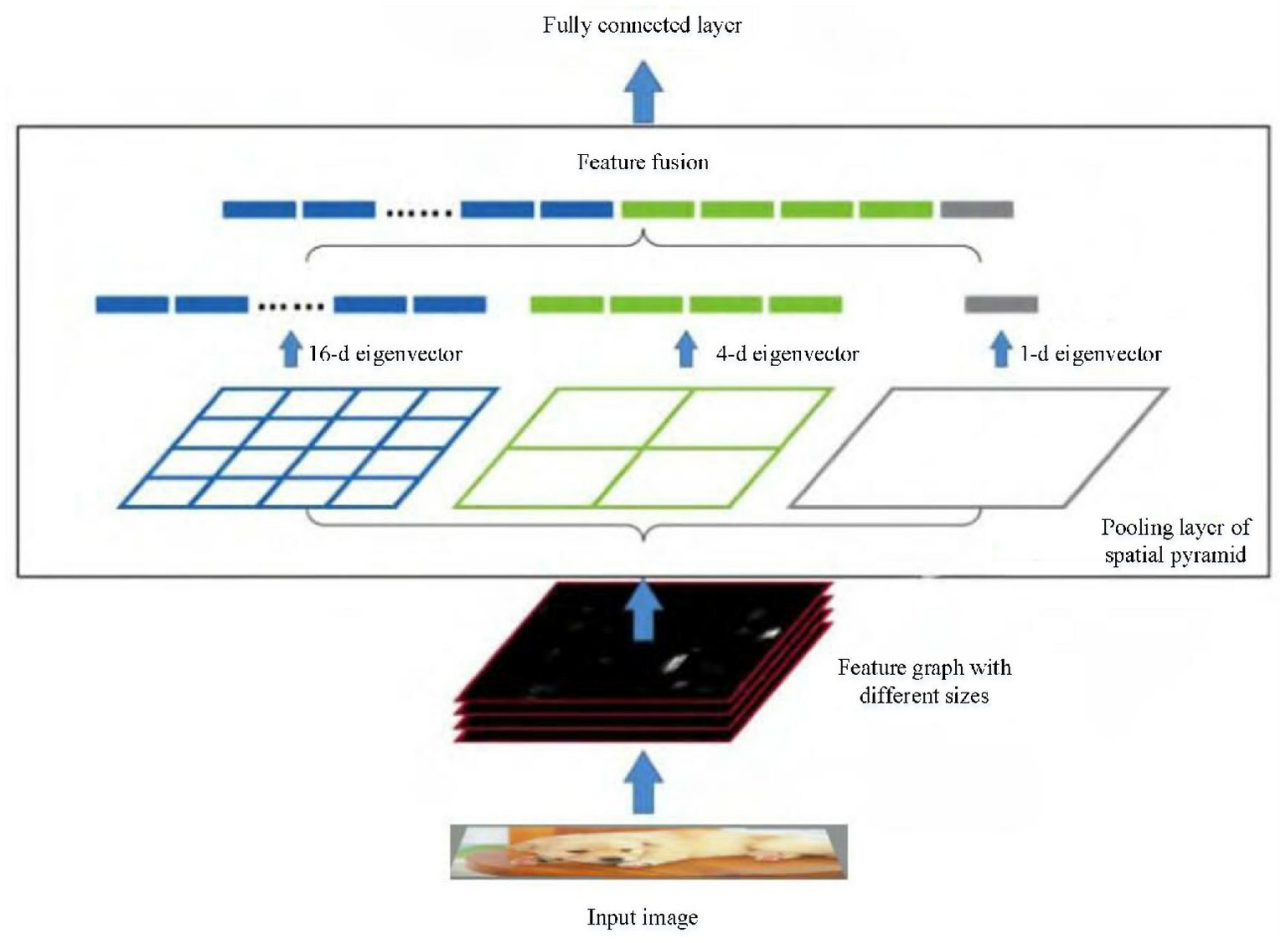
SPP structure.

SPP is essentially multiple average pooling layer. For feature graphs *a* × *a* with different sizes, the output *n* × *n* with fixed size can be generated by adjusting the size and step of slide window. For example, Figure 5 is composed of three average pooling layers. For the input with any size, feature vectors of 4 × 4, 2 × 2 and 1 × 1 are extracted. The feature vectors of 21 dimensions are uniformly output through feature fusion. So that the size of the input image is not limited and the multi-scale semantic features can be extracted.

#### Spatial attention module

In order to highlight significant spatial features, suppress unimportant features and improve the classification accuracy of the network on the basis of increasing the receptive field, parallel input of spatial information and channel information is designed in this paper. The spatial attention module is composed of two 3 × 3 dilated convolution layers and two 1 × 1 convolution layers.

**Figure 6:**
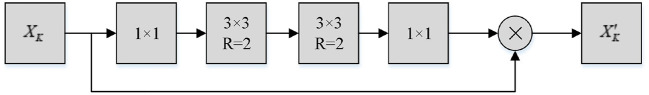
Spatial attention.

The spatial attention module is shown in Figure 6. Firstly, the feature graph is compressed and it passes through two dilated convolution layers with dilated rates 2 and 3, respectively. Finally, the one-dimensional feature graph is generated after compression. The reason for choosing two dilated convolution layers is to maximize the receptive field. However, if only the convolution blocks with large dilated rate are selected, the feature semantics will be spatially discontinuous and the extracted features will be too scattered, which will lead to a decrease rather than an increase in terms of the classification accuracy. Therefore, two 3 × 3 dilated convolution blocks with dilated rates 2 and 3 are selected, so that the two convolution blocks can increase the size of the receptive field and extract continuous feature semantics after superposition. The actual receptive field size of the two dilated convolution layers is:


(13) $$\begin{array}{l} F_{i+1}=F_{i}+\left(k-1\right)\times S_{i} \end{array}$$

Where, *F*_*i*+1_ is the receptive field of the current layer. *F*_*i*_ is the receptive field of the upper layer. *S*_*i*_ is the product of step sizes of all previous layers (excluding current layer). K is the convolution kernel size. After calculation, the actual receptive field size is 12 × 12, represented by *f*^12 × 12^. The output of spatial attention module is:


(14) $$\begin{array}{l} {X^{\prime }}_{K}=X_{K}⊗Ṽ \end{array}$$

Where Ṽ represents the attention graph generated after dilated convolution, and its calculation is as follows:


(15) $$\begin{array}{l} Ṽ=sig{\left(f^{12\times 12}\left(A_{avg}\right)\right)}_{avg} \end{array}$$

Where *A*_*avg*_ is the output of the input feature graph after 1 × 1 convolution. sig is the sigmoid function.

In our proposed CapsNet model, there is a convolution layer with a convolution kernel size of 7 × 7 and step size of 2, which has ReLU non-linear activation function. The second layer is also the convolution layer. The convolution kernel size is 6 × 6 and the step size is 1. Then, a pooling layer is connected, and the pooling core size is 2 × 2, step size is 1, and maximum pooling is adopted. The pooling layer is then connected to a 6 × 6 with step size 1, and the data is reconstructed and then input to the PrimaryCaps layer, so that the main feature information of the image can be obtained as much as possible. Due to the large amount of data, pooling layer is introduced to reduce data dimension to improve model performance and running efficiency.

## Experiments and analysis

The operating system of this algorithm is Windows 10 and GPU is TeslaT4 (64 GB video memory). The deep learning framework adopts PyTorch, and the verification environment is PyCharm+Anaconda.

The experiment data set is cifar-100 benchmark data set. This data set is widely used in the validity of classification algorithms. There are a total of 60,000 color images with size 32 × 32 in the dataset, they are divided into 100 categories, each category contains 600 images. These 100 classes belong to 20 superclasses. In the dataset, 50,000 images are used for training and 10,000 images for testing. The testing parameters are set as follows. The gradient descent method is selected as the optimization algorithm. The batch size is 512. The initial learning rate is 0.1. Training batch size is 100. Momentum is 0.9. The number of iterations is 300. The weight decay is 5 × 10^−4^.

We select four state-of-the-art robot image classification methods [BAVCT (Liu et al., 2022), TRk-CNN (Jun et al., 2021), Feat-WCLTP (Shamaileh et al., 2020)] and the traditional MobileNetV2 to make comparison with proposed method (C-MobileNetV2). The classification effect and error results are shown in Table 3.

**Table 3:** Comparison with different methods.

**Method**	**Classification**	**Error/%**	**Running time/s**
	**accuracy/%**		
MobileNetV2	71.69	1.31	0.55
BAVCT	79.84	0.41	0.88
TRk-CNN	86.71	0.23	0.72
Feat-WCLTP	90.37	0.19	0.61
C-MobileNetV2	96.58	0.02	0.58

The classification accuracy with C-MobileNetV2 is 96.58%, which is improved by 16.74, 9.87, and 6.21% than that of BAVCT, TRk-CNN, Feat-WCLTP, respectively. Also the error is the lowest. The C-MobileNetV2 can achieve better image classification effect. The running time is 0.58 s with C-MobileNetV2, which is higher that MobileNetV2 with 0.55 s due to the introduction of capsule module in MobileNetV2. However, it is lower than that of BAVCT (0.88 s), TRk-CNN (0.72 s), Feat-WCLTP (0.61 s).

In order to verify the designed attention network performance, MobileNetV2 based on capsule network is compared with different classical convolutional neural networks. Four typical neural networks are selected including VGG16 network, ResNet18 residual network, DenseNet networks. The classification effect and improvement effect are shown in Table 4.

**Table 4:** Comparison of classification accuracy of different modules/%.

**Module**	**+SENet**	**+CBAM**	**+ECANet**	**+Capsule**
VGG16	73.24	73.72	73.82	74.24
ResNet18	76.03	76.14	76.30	76.46
DenseNet	75.59	75.90	75.93	76.31
MobileNetV2	75.88	75.96	76.02	76.65

As can be seen from Table 4, for different convolutional neural networks, the classification effect of CBAM module with dual attention mechanism is better than that of SENet module with single attention. The classification accuracy of the newly proposed ECANet module is higher than that of CBAM module, and the C-MobileNetV2 in this paper is better than that of ECANet module.

**Figure 7:**
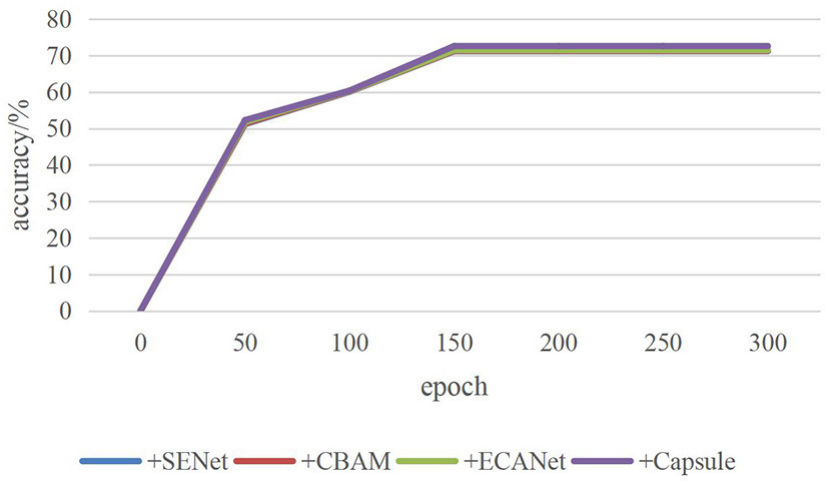
Accuracy change curve.

In the process of 300 iterations, the accuracy changes of the four attention modules in VGG16 are shown in Figure 7. The solid yellow line represents the accuracy changes of C-MobileNetV2 module in each convolutional neural network. It can be seen that the solid yellow line is superior to other colored solid lines in the iterative process. It is similar to ResNet18, DenseNet and MobileNetV2.

In this paper, cross-validation is also used to illustrate the effectiveness of adding attention mechanism module, and the experiment results are shown in Table 5. In here, CAM: channel attention module, SAM: spacial attention module.

**Table 5:** Classification error rates with different improved modules/%.

MobileNetV2	7.11
Capsule	5.54
MobileNetV2+CAM+SAM	5.52
Capsule+CAM+SAM	4.63
MobileNetV2+Capsule (CAM+SAM)	3.78

They denote the best values obtained by proposed method.

Experiment results show that although the traditional MobileNetV2 network has a poor classification effect on the complex data set CIFAR-10, the capsule network with the attention mechanism can improve the accuracy of the complex data set.

The above data analysis effectively proves the effectiveness and superiority of C-MobilenetV2 network, especially the classification accuracy has been greatly improved. The main reasons include two aspects. First, channel attention breaks the shackles of global average pooling and global maximum pooling. It introduces SPP into module, which makes feature extraction more hierarchical, weight distribution more reasonable, and feature characterization ability optimization obvious. Second, the dilated convolution is designed in spatial attention, and the pooling block size and dilated rate size are selected reasonably. Compared with the traditional spatial attention module, C-MobilenetV2 can increase the receptive field, further expand the extraction range and take into account the problem of semantic continuity, achieving a good dynamic balance and extracting saliency feature images more reasonable. The superposition of the optimization effects of the two aspects improves the classification accuracy of the proposed network in this paper significantly.

We also conducts experiments and analysis on high-resolution face age datasets. Face image age estimation has become a very important task in the field of pattern recognition and computer vision, which has a wide range of practical value. Face age estimation is more difficult than the general image classification task because of the small inter-class difference between adjacent ages.

In order to verify the effectiveness of proposed network in high-resolution and challenging image data sets, this paper uses MobilenetV2 as the basic network to conduct experiments on MORPH Album2 and Adience data sets. The experimental results are shown in Tables 6, 7. As can be seen from Table 6, in the MORPH Album2 data set, the MAE value of C-Mobile NETv2 in this paper is significantly reduced. As can be seen from Table 7, in the age group classification comparison experiment conducted on Adience, an unrestricted age data set, the age estimation accuracy obtained by C-MobilenetV2 is higher than the classification accuracy of MobilenetV2 network at the same level. The experimental results show that C-MobilenetV2 network has better learning ability than the original MobilenetV2 network on MORPH Album2 and Adience high resolution data sets, which verifies the adaptability of C-MobilenetV2 in different types and different resolutions of data sets.

**Table 6:** Experimental results of different models on MORPH Album2.

**Model**	**MAE**
MobilenetV2	3.52
C-MobilenetV2	**3.05**

They denote the best values obtained by proposed method.

**Table 7:** Experimental results of different models on Adience/%.

**Model**	**Average accuracy**	**1-off**
MobilenetV2	68.67	93.43
C-MobilenetV2	**72.98**	**93.85**

They denote the best values obtained by proposed method.

## Conclusion

The MobileNetV2 combined with capsule network model can effectively solve the problems of insufficient feature extraction of original MobileNetV2 network and poor performance in complex data sets. In the feature extraction process, we first adopt capsule network to obtain feature graph. Based on the lightweight MobileNetV2 network, a channel-space dual collaborative attention module is designed, which is embedded in MobileNetV2 network structure and successfully applied to image classification. On the CIFAR-100 public standard data set, different classical convolutional neural networks and different attention modules are embedded for comparison, which achieves good results and greatly improves the classification accuracy, it can fully confirm the effectiveness of the proposed algorithm. Future works will be researched in the area of image classification with advanced deep learning methods.

## Data availability statement

The original contributions presented in the study are included in the article/supplementary material, further inquiries can be directed to the corresponding author.

## Author contributions

JZ implemented the code and draft the manuscript. XY and XL assisted to implement the code and discussed the manuscript. CW guided the research and discussed the results. JZ guided the research, implemented parts of code, and revised the manuscript. All authors contributed to the article and approved the submitted version.

## Funding

This work was supported in part by the National Natural Science Foundation of China under Grant Nos. U20A20197, 61973063, U1713216, 61901098, and 61971118, Liaoning Key Research and Development Project 2020JH2/10100040, the China Postdoctoral Science Foundation 2020M670778, the Northeastern University Postdoctoral Research Fund 20200308, the Scientific Research Foundation of Liaoning Provincial Education Department LT2020002, the Foundation of National Key Laboratory (OEIP-O-202005), and the Fundamental Research Fund for the Central Universities of China N2026005, N181602014, N2026004, N2026006, N2026001, and N2011001.

## Conflict of interest

The authors declare that the research was conducted in the absence of any commercial or financial relationships that could be construed as a potential conflict of interest.

## Publisher's note

All claims expressed in this article are solely those of the authors and do not necessarily represent those of their affiliated organizations, or those of the publisher, the editors and the reviewers. Any product that may be evaluated in this article, or claim that may be made by its manufacturer, is not guaranteed or endorsed by the publisher.
